# A 45-year old man with recurrent syncope: an unusual presentation of coronary artery disease

**DOI:** 10.11604/pamj.2013.14.71.2166

**Published:** 2013-02-19

**Authors:** Abiodun Moshood Adeoye, Aina Nnodim Adekunle, Adewole Adesoji Adebiyi, Ajit Mullassari, Subban Vijayakumar, Chibuike Eze Nwafor

**Affiliations:** 1 Cardiology Unit, Department of Medicine, University College Hospital, Ibadan Nigeria; 2 Department of Medicine, College of Medicine, University of Ibadan, Nigeria; 3 Institute of Cardiovascular Diseases, Madras Medical Mission, Chennai India

**Keywords:** Syncope, coronary artery disease, angiogram, Percutaneous Transluminal Coronary Angioplasty

## Abstract

A 45-year old normotensive, euglycaemic, non-smoker was referred from a peripheral hospital to the Cardiology unit of the University College Hospital, Nigeria for evaluation of recurrent exercise induced syncope. Initial 12-lead electrocardiogram (ECG), 24-hr ambulatory ECG, trans-thoracic echocardiogram and electroencephalogram (EEG) were normal. A repeat episode of syncope warranted further investigation. Immediate post syncope ECG showed deeply inverted symmetrical T waves in the anterior leads. He underwent coronary angiogram which revealed distal left main disease and 70-80% stenosis of the proximal Left Anterior Descending Artery (LAD). The Circumflex artery was non dominant with normal Right Coronary artery. He subsequently had Percutaneous Transluminal Coronary Angioplasty (PTCA) of the LAD. Post-revascularisation course has been satisfactory with no recurrence of syncope. In view of the rising trend of cardiac death in the country, there is the need for high index of suspicion in making diagnosis of coronary artery disease in patients with syncope.

## Introduction

Coronary artery disease had historically been considered to be uncommon in the sub-Saharan African of Negro origin[1, 2]. In Nigeria, Annual Medical and Sanitary Reports (AMSR) sent regularly to the Colonial Secretary in London by the colonial government showed that coronary heart disease was distinctly rare from 1898 to 1960. Subsequent studies though hospital-based also buttressed the rarity of coronary artery disease in Nigeria[2]. However more recent studies have demonstrated the trend of a rising prevalence of coronary artery disease (CAD) in sub-Saharan Africa [3–6]. The epidemiology of the traditional risk factors of coronary artery disease has equally been well-studied [7–9]. While clinical manifestations of CAD among white population are well documented in literature with the pathognomonic exertional chest pains relieved by nitrates or rest, and angina equivalents which include dyspnoea, dyspepsia[10], very few studies on manifestations of coronary artery disease in the black African are available[7]. We report an unusual presentation of coronary artery disease in a black African man with few risk factors for coronary artery disease.

## Patient and observation

A 45-year old normotensive, euglycaemic, non-smoker was referred from a peripheral hospital to the Cardiology unit of the University College Hospital, Nigeria for evaluation of a syncopal attack. He was in apparent good health until 2 weeks before presentation when he suddenly felt severe giddiness, not preceded by chest pains, palpitations or breathlessness. He was admitted at the peripheral hospital for two days during which the giddiness resolved and was subsequently discharged with instruction to exercise regularly. The first exercise of brisk walk for about thirty minutes in company of a friend was not eventful; however a repeat exercise the following day resulted in a syncopal episode lasting few minutes. He sustained no head injury and there were no associated seizures or urinary incontinence. He was subsequently referred to our centre for further evaluation. Past medical history was unremarkable except for an appendicectomy in 1985 and a urological procedure in 2001 during investigation for infertility. He had no adverse reactions or sequelae.

Examination revealed a conscious middle aged man who was not in any distress. He was neither pale nor cyanosed and had no pedal oedema. His pulse was 60 beats per min, regular and normal volume. The peripheral pulses were palpable and there was no carotid bruit. Blood pressure was 130/90 mmHg. First and second heart sounds only were heard. The respiratory, abdominal and nervous system examinations were not remarkable.

**Figure 1:**
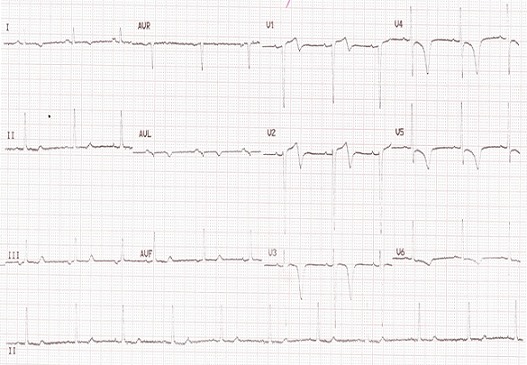
Immediate post syncope 12-lead ECG of the patient

Initial resting 12-lead ECG, 24-hr ambulatory ECG, trans-thoracic echocardiogram and electroencephalogram (EEG) were essentially normal. Another episode of syncope warranted further investigation. Immediate post syncope ECG showed deeply inverted symmetrical T waves in the anterior leads (Figure 1).

An assessment of ischaemic heart disease was made and he was referred to the Madras Medical Mission Chennai India for Coronary angiography and possible revascularisation.

**Figure 2:**
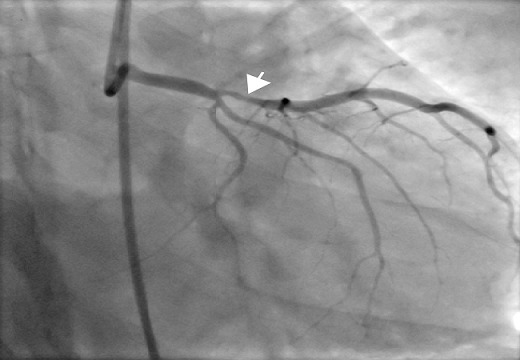
Coronary angiogram with a stenosis of the LAD (arrow)

On admission at the centre, chest X-ray revealed no abnormalities. Resting ECG showed sinus bradycardia with a normal QRS axis and T wave inversion in leads V1-V5. Trans-thoracic echocardiogram showed no regional wall motion abnormalities, normal LV ejection fraction, type II diastolic dysfunction, concentric LVH, mild tricuspid regurgitation and pulmonary arterial hypertension (PAH). CK-MB was 17IU/L and troponin I was 0.095ng/ml. Coronary angiogram revealed 70-80% stenosis in the ostial-proximal LAD (Figure 2). Normal right coronary artery with non dominant circumflex artery.

**Figure 3:**
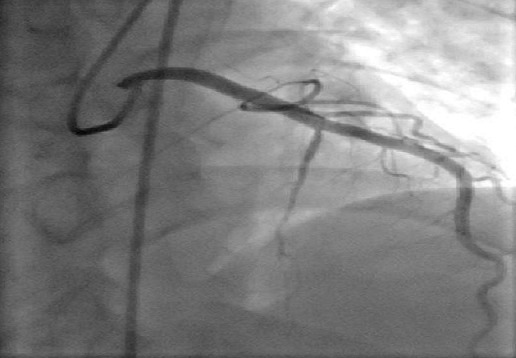
Post PTCA coronary angiogram showing adequate flow in the LAD

He had a successful PTCA + DES stent to the LAD (Figure 3). He was also treated with antiplatelets, statins and low molecular weight heparin. Post-procedure course was uneventful and he was discharged after 3 days with stable vital signs.

## Discussion

The traditional risk factors for atherosclerotic coronary artery disease are widely recognised. The non-modifiable risk factors include age >65 years, male sex, and a family history of coronary artery disease. The modifiable risk factors include diabetes, hypertension, dyslipidaemia, smoking history, obesity, sedentary lifestyle and elevated homocysteine.

There was paucity of these traditional risk factors in the case being presented, gender and low HDL-C being the only definite risk. Also the typical clinical manifestation of atherosclerotic coronary artery disease, angina pectoris, was significantly absent in the case presented. Epidemiologically, silent myocardial infarctions have been found to be more prevalent in diabetics. In the elderly atypical presentations of coronary artery disease may be seen - dyspepsia, dyspnoea, nausea and vomiting, termed angina equivalents. Syncope and presyncope in isolation are uncommon modes of presentation of coronary artery disease.

Syncope is a symptom, defined as a transient, self limiting loss of consciousness with a relatively rapid onset and usually leading to fall; the subsequent recovery is spontaneous, complete, and usually prompt. The underlying mechanism is a transient global cerebral hypoperfusion. Clinical features suggestive of a cardiac syncope include occurrence during exertion or in the supine position, association with palpitations or chest pains, or occurrence on the background of demonstrable structural heart disease, all of which were absent in our patient. Holter ECG also failed to demonstrate any arrhythmias.

In the case being presented, despite the benign appearance of the syncopal episodes, and absence of classical symptoms of coronary artery disease, the deeply inverted T-wave in the anterior leads which were symmetrical was worrisome and necessitated referral to Madras Medical Mission for further management.

## Conclusion

In view of the rising trend of cardiac death in the country, there is the need for high index of suspicion in making diagnosis of coronary artery disease in patients with recurring syncope.
